# Effect of Late Testing and Antiretroviral Treatment on Mortality Among People Living With HIV in the Era of Treat-All in Guangdong Province, China, 1992–2018: A Cohort Study

**DOI:** 10.3389/fpubh.2022.851117

**Published:** 2022-07-08

**Authors:** Qiaosen Chen, Jun Liu, Xiaobing Fu, Fang Yang, Qicai Liu, Junbin Li, Zhimin Tan, Jing Li, Kaihao Lin, Yao Yan, Yi Yang, Yan Li, Hongbo Jiang

**Affiliations:** ^1^Department of Epidemiology and Biostatistics, School of Public Health, Guangdong Pharmaceutical University, Guangzhou, China; ^2^Department of HIV/AIDS Control and Prevention, Guangdong Provincial Center for Disease Control and Prevention, Guangzhou, China; ^3^Guangzhou Eighth People's Hospital, Guangzhou Medical University, Guangzhou, China

**Keywords:** people living with HIV, late testing, late antiretroviral treatment, treat-all, mortality, trend

## Abstract

Late testing and antiretroviral therapy (ART) prevailed among people living with HIV (PLHIV) and impacted the benefit of immediate ART. This study aimed to identify the benefit of the test-and-immediate-treat policy in China, the effect of immediate ART, and the influence of late testing and ART on the whole PLHIV in Guangdong Province, China. We designed two tendency analyses in aggregative form and two cohorts (surveillance and ART cohort) in individuals' perspectives based on the HIV/AIDS Comprehensive Response Information Management System. Two interrupted time series models were conducted for tendency analysis from 2009 to 2018 to explore the all-cause and short-term mortality decrease after the test-and-immediate-treat policy. A time-dependent Cox model was performed for the surveillance cohort from 1992 to 2018 and a joint model was utilized for the ART cohort to identify the effect of immediate ART and the influence of late testing and ART on death. The tendency analysis included 324,914 and 68,679 person-year for all-cause/short-term mortality. A total of 49,289 and 26,287 PLHIV were recruited in the surveillance and ART cohort with 5,557 and 459 deaths, respectively. The short-term mortality dropped from 4.69 cases/100 person-year in January 2009 to 0.35 cases/100 person-year in December 2018 (standardized rate). The all-cause mortality saw a decreasing trend from 1.46 cases/100 person-year in January 2009 to 0.14 cases/100 person-year in December 2018 (standardized rate). The tendency analysis showed a significant short-term mortality slope decrease after the test-and-immediate-treat policy (*P* = 0.024). From the surveillance cohort, late testing, in general, was a risk factor for all-cause mortality [*hazard ratio* (*HR)* = 1.330, 95% *CI*, 1.250, 1.416]. ART cohort showed higher hazards of all-cause mortality among PLHIV with no late testing, but late ART (*HR* = 1.690, *95% CI*, 1.166, 2.451) and both the late testing and late ART (*HR* = *1.335, 95% CI, 1.042, 1.710*). Immediate ART might decrease the hazard of all-cause death though it is insignificant (*HR* = 0.923, 95% CI: 0.755, 1.129) in the ART cohort. The test-and-immediate-test policy brought benefit to PLHIV. We should enlarge HIV testing using comprehensive approaches to decrease late testing and ART and increase the benefit of immediate ART.

## Introduction

Tremendous efforts have been made to the progress of the HIV care continuum around the world. However, it is still insufficient; by 2020, very few low- and middle-income countries (LMICs) can achieve 90-90-90 ambition (1). One of the essential obstacles is undiagnosed people living with HIV (PLHIV). According to the Joint United Nations Programme on HIV/AIDS (UNAIDS) 2020 data, apart from Australia, all the other Asian and Pacific countries included could not reach the first 90 targets that 90% of PLHIV who know their status (1). Unawareness of HIV infection is a lingering concern, rendering a disadvantaged position of late testing and antiretroviral therapy (ART), sequentially leading to a higher mortality rate.

Albeit difficulties in HIV diagnosis coverage, we have accustomed a significant step into ART coverage. The UNAIDS 2015 appealed for treat-all and many countries followed the recommendation well. China responded quickly, formalizing the test-and-immediate-treat guideline after 1 year of the UNAIDS appeal (2). Such international cooperation and national effort should be appreciated, sacrificing massive recourse to enlarge the immediate ART coverage.

Immediate ART prevailed in China after the implementation of the test-and-immediate-treat policy. The recent national cohort in China showed that immediate ART significantly improved prognosis among PLHIV with relatively high CD4 (3). From the evidence in high-income settings, those with CD4 < 200 cells/μl appeared to gain much less from the immediate ART (4). However, until now, we had no evidence of whether the treat-all policy in China received the health benefit for the whole PLHIV in the population perspective. In addition, few studies explored how late testing and late ART influenced immediate ART in LMIC and how to gain more from the treat-all policy.

In this study, we aimed to evaluate the effect of the test-and-immediate-treat policy, explore the influence of late testing and late ART on all-cause mortality in the era of immediate ART, and put forward the pragmatic suggestion for future HIV control and prevention in Guangdong Province, China.

## Materials and Methods

### Study Setting and Design

Guangdong Province, with more than 0.1 billion registered population, is among the top 5 provinces in the number of PLHIV in China. As the most developed province in China, every year, a lot of internal migrants go to Guangdong and worked there.

The data of this study were collected through the HIV/AIDS Comprehensive Response Information Management System (CRIMS). The tendency analyses were designed based on the CRIMS. The surveillance cohort was conducted based on the CRIMS (5) to study the mortality among patients with confirmed HIV diagnosis from 1992 to 2018 in Guangdong province, China. The ART cohort was generated by linking the surveillance cohort with the ART for adults system (5). The detail of the subsystems in China has been described elsewhere (5).

This study recruited all the registered PLHIV in Guangdong Province, China, and, thus, we did not need to estimate the sample size.

### Enrollment

Tendency analysis recruited all the 15-year-old and above participants whose date of HIV diagnosis and date of mortality or the date of the last follow-up could be collected. We summarize the tendency data in an aggregated form.

**Figure 1:**
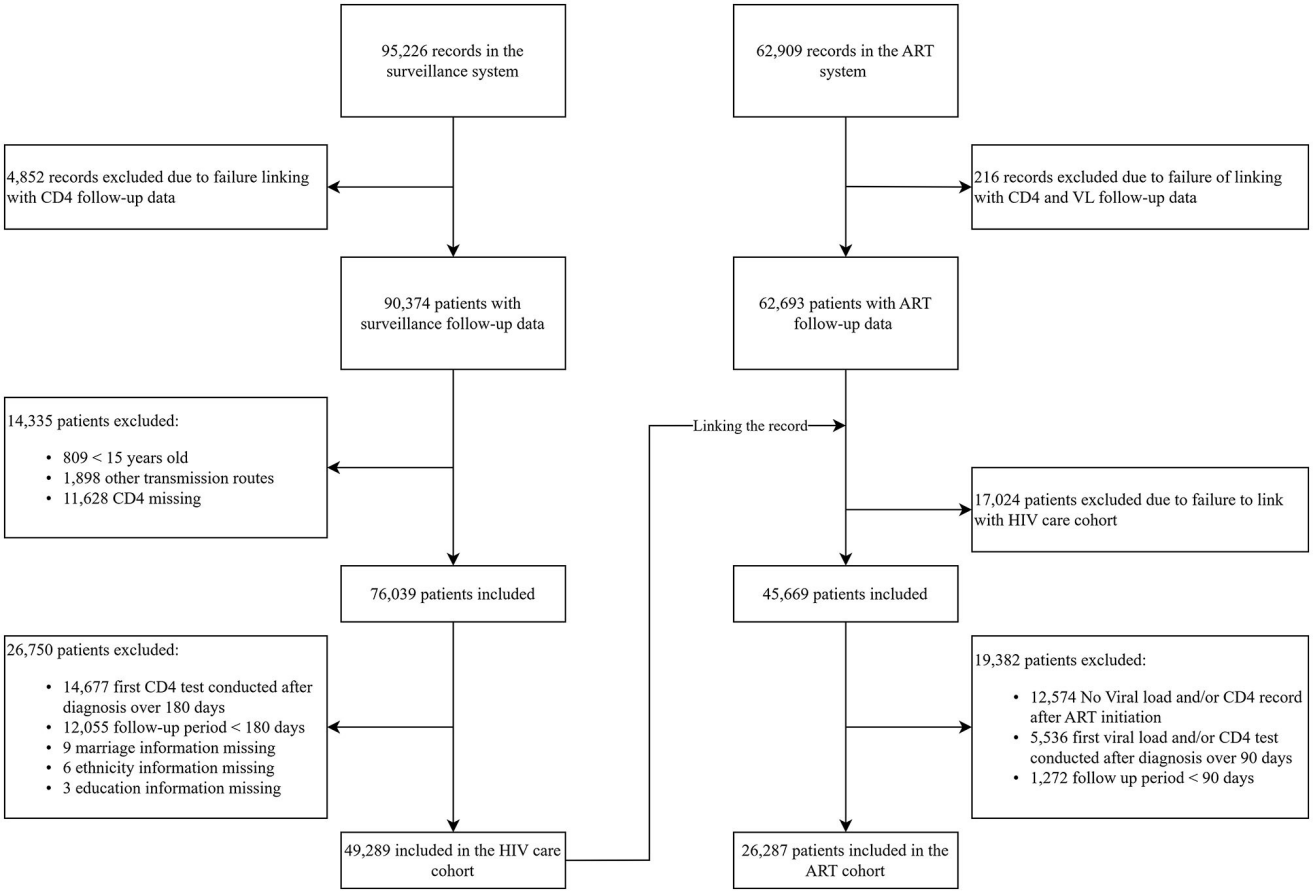
Flowchart showing the design of surveillance and the antiretroviral therapy (ART) cohort.

The surveillance cohort recruited the participants from 1992 to 2018 if they reached the following criterion: (1) age of 15-year-old or above; (2) get HIV infection *via* sexual intercourse or injection drug use (IDU); (3) first CD4 cell count test within 180 days of HIV diagnosis; (4) follow-up period more than 180 days; and (5) no missing data in demographics. Individuals who acquired HIV through sexual contact and IDU accounted for over 90% of newly diagnosed HIV infections during this time (6). The ART cohort recruited the participants if they reached the inclusion criterion of the surveillance cohort: (1) under the test of viral load and CD4 after ART; (2) first viral load and CD4 test conducted over 90 days of ART initiation; and (3) follow-up period more than 90 days after ART initiation (details of enrollment are shown in Figure 1).

### Definitions

Advanced HIV disease (AHD) status was defined based on two indicators, namely, late testing and late ART. Late testing refers to PLHIV presenting for care with a CD4 < 200 cells/μl or presenting with an AIDS-defining event, regardless of the CD4 cell count within 6 months after diagnosis (6). Late ART refers to PLHIV initiating ART without CD4 < 200 cells/μl and AIDS diagnosis over 90 days (7). Immediate ART refers to PLHIV initiating ART within 30 days after HIV diagnosis (8). Short-term death refers to that participants dying within 1 year after HIV diagnosis regardless of the cause of death (9).

### Predictors

In this study, we took the following predictors, which direct or intermediate influence mortality into account: age, sex, ethnicity, marriage, education, transmission, detection sources, CD4 cell counts, viral load (VL), early/late testing, early/late ART and immediate/delayed ART, ART interruption or discontinuation, and polypill regimen.

### Outcomes

The primary outcome was all-cause mortality. For tendency analysis, we analyzed short-term mortality as well.

### Statistical Analysis

We first conducted an interrupted time series analysis with a segment log-linear model to explore the pre- and post-intervention effect and a corresponding difference in the test-and-immediate-treat policy using aggregative data using the 10-year mortality surveillance. We considered the epidemiological transition and standardized the rate by age and sex stratification in 2018. China implemented the policy in mid-year 2016, which requires time for health sectors to accustom. We, therefore, delayed half of the year and set the dummy variable in January 2017 to distinguish between before and after the policy. To examine the fit of the log-linear model, we used a flexible locally weighted regression (LOESS) model and compared the predicted value between the two models.

A time-dependent Cox model was performed to study the relationship between demographics, clinical and laboratory measurements, and all-cause mortality in the surveillance cohort where the model incorporated repeated measurement of CD4 cell count. After linking the surveillance cohort treatment information, we applied a joint model to evaluate the hazard ratio (HR) between late testing/ART and immediate ART and all-cause mortality among the PLHIV receiving ART from 2005 to 2018. The joint model requires two submodels, including the generalized mixed linear model and a time-to-event model, where we specified the log_10_ viral load trajectory by a linear mixed model and the time-to-event process was described by a Cox model to study the relationship between late testing/ART and immediate ART and all-cause mortality after adjusting demographics.

In the longitudinal process, we modeled the trajectory of VL rather than that of CD4 because, according to the WHO monitoring guideline, VL monitoring is preferred and it is reasonable to stop CD4 monitoring for the stable patients in the setting of viral load monitoring routine available (10). Secondary, in a particular setting such as HIV and human T-cell lymphotropic virus type 1 (HTLV-1) coinfection, contrary to the scientific common sense, the increase of CD4 links with a higher risk of death (11). We failed to consider it due to no related information collected in the CRIMS. In addition, late testing and late ART are two predictors generated partially based on CD4 and also the model for the time-to-event process has incorporated these two predictors. If CD4 was incorporated in the longitudinal process together, it might be redundant for model construction. Therefore, the VL submodel finally incorporated basic demographics for adjustment and more clinical variables to predict VL better such as clinical practitioners' advice on ART continuation status. Of note, the VL model did not incorporate the variable of “immediate ART” because before the test-and-immediate-treat policy, clinical practitioners were more likely to suggest ART to those with high VL (Supplementary Table 1). Incorporating the variable of “immediate ART” in the VL submodel had a risk of ignoring temporal relations, which might bring some difficulties in interpretation and model construction. We additionally performed a subgroup analysis because the web-based national HIV/AIDS information system in China was built well in 2008 (5) and meanwhile China replaced the previous ART recommendation for PLHIV with CD4 < 200 cells/μl or AIDS by PLHIV with CD4 < 350 cells/μl or AIDS during 2008. To minimize the influence of changes in treatment initiation guidelines and system building, we restricted the subpopulation of PLHIV initiating ART after 2009 and conduct a subgroup analysis from 2009 to 2018.

We performed interrupted time series analysis in Stata/IC 16 (serial number: 301606333568, license to Qiao Sen Chen) using the itsa module (12) and another analysis was conducted in R software using the JM package (13). In this study, all the tests are two-sided with a significant level of 0.05.

### Ethical Considerations

The Ethics Committee of Guangdong Pharmaceutical University approved the implementation of this study. All the participants signed an informed consent when recorded in the HIV/AIDS case reporting system. In this study, we deidentified all the participants before analysis.

## Results

### Tendency for Mortality From 2009 to 2018

**Figure 2:**
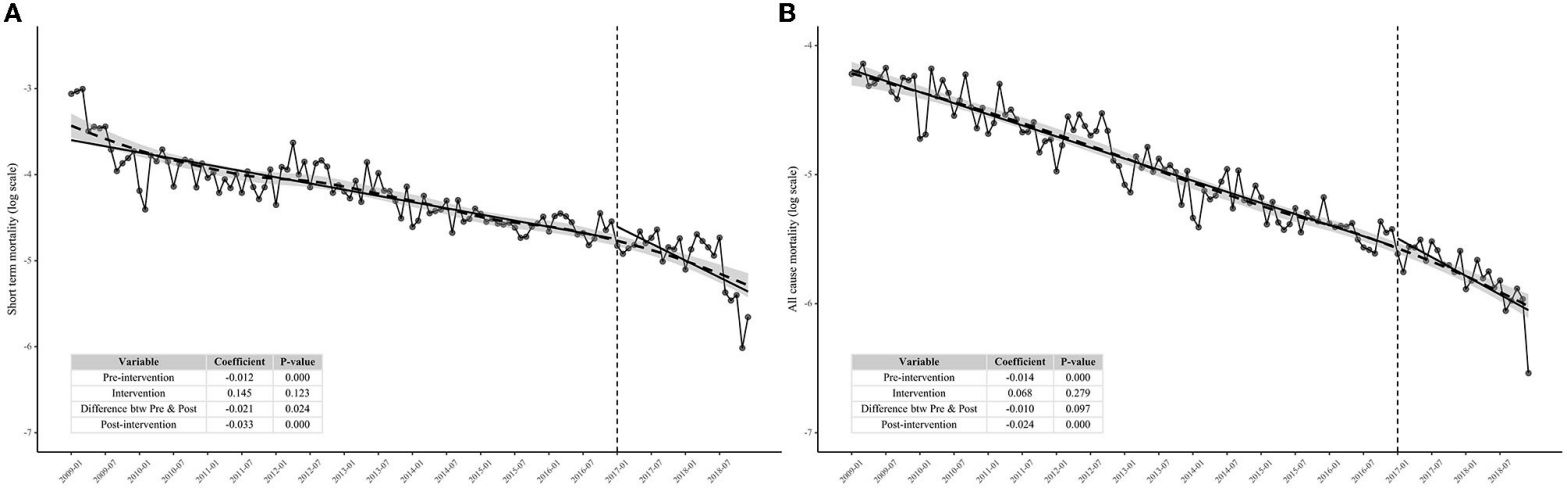
Interrupted time series analysis showing the effect of test-and-immediate-treat policy on short-term mortality and all-cause mortality among people living with HIV in Guangdong Province from 2009 to 2018. **(A)** Effect of test-and-immediate-treat policy on short-term mortality and **(B)** effect of test-and-immediate-treat policy on all-cause mortality.

We conducted two 10-year follow-up (from 2009 to 2018) mortality tendency analyses, covering 324,914 person-year and 19,259 cases of all-cause death for all-cause mortality analysis and 68,679 person-year and 8,667 cases of short-term death for short-term mortality analysis. Short-term mortality dropped from 4.69 cases/100 person-year in January 2009 to 0.35 cases/100 person-year in December 2018 (standardized rate). Meanwhile, the all-cause mortality saw a decreasing trend from 1.46 cases/100 person-year in January 2009 to 0.14 cases/100 person-year in December 2018 (standardized rate). As for short-term mortality, it is a significant difference in decrease between preintervention and postintervention, showing that the test-and-immediate-treat policy has accelerated the decline of short-term mortality (*P* = 0.024, Figure 2A), whereas, after 2 years of surveillance, we could not observe the appreciable mortality decrease difference between preintervention and postintervention yet, but all-cause mortality still decreased steadily during the two years (*P* = 0.097, Figure 2B). Figure 2 shows that the fit by LOESS (dashed line) has a very similar predicted curve to that by the segment log-linear model (solid line), which verified the goodness of fit by the segment log-linear model.

### Characteristics of the Surveillance Cohort and the Antiretroviral Therapy Cohort

In total, 49,289 and 26,287 PLHIV were recruited in the surveillance cohort and the ART cohort with 5,557 and 459 deaths, respectively. According to Tables 1, 2, CD4 at entry was 317.33 and 323.55 cells/μl in the surveillance and ART cohort separately, but the difference enlarged at the end of follow-up where CD4 at the surveillance cohort is 431.61 cells/μl and that at the ART cohort is 464.91 cells/μl. In the ART cohort, 46.2% of PLHIV with VL > 400 copies/ml at the entry and the proportion reduced to 4.5% at the end of the cohort. Less than half (45.4%) of PLHIV can receive immediate ART. A total of 56% of PLHIV were identified as neither late testing nor late ART and 36.2% were PLHIV with both late testing and late ART.

**Table 1:** Characteristics of people living with HIV recruited in the surveillance cohort.

**Characteristics**	**Alive (%)**	**Death (%)**	**Overall (%)**
**Total**	43732 (100.0)	5557 (100.0)	49289 (100.0)
**Age, yrs**			
15~29	16295 (37.3)	874 (15.7)	17169 (34.8)
30~44	17817 (40.7)	2206 (39.7)	20023 (40.6)
45~59	6988 (16.0)	1138 (20.5)	8126 (16.5)
60~	2632 (6.0)	1339 (24.1)	3971 (8.1)
**Sex**			
Female	8286 (18.9)	868 (15.6)	9154 (18.6)
Male	35446 (81.1)	4689 (84.4)	40135 (81.4)
**Ethnicity**			
Han	41251 (94.3)	5282 (95.1)	46533 (94.4)
Others	2481 (5.7)	275 (4.9)	2756 (5.6)
**Marital status**			
Single	19764 (45.2)	1682 (30.3)	21446 (43.5)
Married or with partner	17220 (39.4)	2497 (44.9)	19717 (40.0)
Widowed or divorced	6515 (14.9)	1309 (23.6)	7824 (15.9)
Unknown	233 (0.5)	69 (1.2)	302 (0.6)
**Educational attainment**			
Tertiary education and above	8586 (19.6)	166 (3.0)	8752 (17.8)
Senior high school	10003 (22.9)	574 (10.3)	10577 (21.5)
Junior high school	17629 (40.3)	2444 (44.0)	20073 (40.7)
Primary school	6770 (15.5)	2040 (36.7)	8810 (17.9)
Illiteracy	744 (1.7)	333 (6.0)	1077 (2.2)
**Transmission**			
Homosexual intercourse	15968 (36.5)	380 (6.8)	16348 (33.2)
Heterosexual intercourse	24342 (55.7)	3515 (63.3)	27857 (56.5)
Injection drug use	3226 (7.4)	1584 (28.5)	4810 (9.8)
Sexual intercourse + injection drug use	196 (0.4)	78 (1.4)	274 (0.6)
**Occupation**			
Farmer/worker	7780 (17.8)	1984 (35.7)	9764 (19.8)
Business/service	18783 (43.0)	1775 (31.9)	20558 (41.7)
Professional/manager	1838 (4.2)	297 (5.3)	2135 (4.3)
Others	15331 (35.1)	1501 (27.0)	16832 (34.1)
**Source of detection**			
Medical institution	24548 (56.1)	3217 (57.9)	27765 (56.3)
Voluntary counseling and testing	12414 (28.4)	802 (14.4)	13216 (26.8)
Special project	2509 (5.7)	78 (1.4)	2587 (5.2)
Others	4261 (9.7)	1460 (26.3)	5721 (11.6)
**Internal migrant**			
Yes	10853 (24.8)	1769 (31.8)	12622 (25.6)
No	32879 (75.2)	3788 (68.2)	36667 (74.4)
**Late testing**			
Yes	14034 (32.1)	2617 (47.1)	16651 (33.8)
No	29698 (67.9)	2940 (52.9)	32638 (66.2)
**CD4 at entry to surveillance cohort, mean(sd) (cells/μL)**	323.89 (204.18)	265.72 (214.90)	317.33 (206.24)
**Most recent CD4, mean(sd) (cells/μL)**	455.36 (213.90)	244.73 (204.69)	431.61 (223.06)
**Times of CD4 test, mean(sd)**	9.16 (6.26)	4.97 (3.74)	8.68 (6.17)

*sd, standard deviation*.

**Table 2:** Characteristics of people living with HIV recruited in the ART cohort.

**Characteristics**	**Alive (%)**	**Death (%)**	**Overall (%)**
**Total**	25828 (100.0)	459 (100.0)	26287(100.0)
**Age, yrs**			
15~29	8037 (31.1)	32 (7.0)	8069 (30.7)
30~44	11913 (46.1)	143 (31.2)	12056 (45.9)
45~59	4386 (17.0)	126 (27.5)	4512 (17.2)
60~	1492 (5.8)	158 (34.4)	1650 (6.3)
**Sex**			
Female	4497 (17.4)	91 (19.8)	4588 (17.5)
Male	21331 (82.6)	368 (80.2)	21699 (82.5)
**Ethnicity**			
Han	24639 (95.4)	452 (98.5)	25091 (95.5)
Others	1189 (4.6)	7 (1.5)	1196 (4.5)
**Marital status**			
Single	12094 (46.8)	74 (16.1)	12168 (46.3)
Married or with partner	9916 (38.4)	251 (54.7)	10167 (38.7)
Widowed or divorced	3697 (14.3)	130 (28.3)	3827 (14.6)
Unknown	121 (0.5)	4 (0.9)	125 (0.5)
**Educational attainment**			
Tertiary education and above	6392 (24.7)	24 (5.2)	6416 (24.4)
Senior high school	6579 (25.5)	62 (13.5)	6641 (25.3)
Junior high school	9626 (37.3)	185 (40.3)	9811 (37.3)
Primary school	2899 (11.2)	159 (34.6)	3058 (11.6)
Illiteracy	332 (1.3)	29 (6.3)	361 (1.4)
**Transmission route**			
Homosexual intercourse	11114 (43.0)	22 (4.8)	11136 (42.4)
Heterosexual intercourse	13546 (52.4)	343 (74.7)	13889 (52.8)
Injection drug use	1168 (4.5)	94 (20.5)	1262 (4.8)
**Occupation**			
Farmer/worker	3524 (13.6)	160 (34.9)	3684 (14.0)
Business/service	11678 (45.2)	149 (32.5)	11827 (45.0)
Professional/manager	1301 (5.0)	57 (12.4)	1358 (5.2)
Others	9325 (36.1)	93 (20.3)	9418 (35.8)
**Source of detection**			
Medical institution	14740 (57.1)	319 (69.5)	15059 (57.3)
Voluntary counseling and testing	7895 (30.6)	84 (18.3)	7979 (30.4)
Special project	1664 (6.4)	4 (0.9)	1668 (6.3)
Others	1529 (5.9)	52 (11.3)	1581 (6.0)
**Internal migration**			
No	22342 (86.5)	425 (92.6)	22767 (86.6)
Yes	3486 (13.5)	34 (7.4)	3520 (13.4)
**CD4 at entry to ART cohort, mean (sd), cells/μL**	325.58 (197.07)	209.83 (169.94)	323.55 (197.21)
**Most recent CD4, mean (sd), cells/μL**	469.21 (220.83)	223.40 (173.94)	464.91 (222.43)
**VL at entry to ART cohort, copies/ml**			
≤ 400	13870 (53.7)	260 (56.6)	14130 (53.8)
>400	11958 (46.3)	199 (43.4)	12157 (46.2)
**Most recent viral load, copies/ml**			
≤ 400	24818 (96.1)	287 (62.5)	25105 (95.5)
>400	1010 (3.9)	172 (37.5)	1182 (4.5)
**Times of CD4 and/or VL, mean (sd)**	4.51 (3.81)	3.27 (1.95)	4.49 (3.79)
**AHD status**			
Neither late testing nor late ART	14593 (56.5)	118 (25.7)	14711 (56.0)
Late testing but not late ART	608 (2.4)	18 (3.9)	626 (2.4)
Late ART but not late testing	1400 (5.4)	44 (9.6)	1444 (5.5)
Both late testing and late ART	9227 (35.7)	279 (60.8)	9506 (36.2)
**Immediate ART**			
No	14077 (54.5)	275 (59.9)	14352 (54.6)
Yes	11751 (45.5)	184 (40.1)	11935 (45.4)
**Clinical decision to ART continuation**			
ART regimen switch	7787 (30.1)	185 (40.3)	7972 (30.3)
Keeping ART regimen unchanged	17146 (66.4)	218 (47.5)	17364 (66.1)
ART discontinuation	895 (3.5)	56 (12.2)	951 (3.6)

*sd, standard deviation; VL, viral load; ART, antiretroviral therapy; AHD, advanced HIV disease*.

### Factors Associated With All-Cause Mortality in the Surveillance Cohort

Older people were more likely to die in the surveillance cohort than 15- to 29-year-old PLHIV. Females were less likely to die and lower education attainment was linked with more probability of death than the group of tertiary education and above. Patients getting HIV *via* heterosexual intercourse and IDU had a higher mortality rate compared to those *via* homosexual intercourse. Individuals tested through voluntary counseling and testing (VCT) programs were associated with a lower death rate than those tested from medical institutions. Internal migrants had a higher HR of death. Lower CD4 at entry and late testing both indicated a higher mortality rate in general (Table 3).

**Table 3:** Risk factors summary of all-cause death from the extended Cox regression and joint modeling in the surveillance and ART cohorts.

	**HR (95% CI) of all-cause death in the surveillance cohort (1992–2018)**	**HR (95% CI) of all-cause death in the surveillance cohort** ** (2009–2018)**	**HR (95% CI) of all cause death in the ART cohort (2005–2018)**	**HR (95% CI) of all-cause death in the ART cohort** ** (2009–2018)**
**Age, yrs**				
15~29	ref.	ref.	ref.	ref.
30~44	1.494 (1.376, 1.623)	1.51 (1.388, 1.642)	1.231 (0.816, 1.855)	1.215 (0.808, 1.826)
45~59	2.617 (2.368, 2.892)	2.691 (2.433, 2.977)	2.61 (1.692, 4.027)	2.57 (1.669, 3.959)
60~	4.85 (4.314, 5.453)	4.988 (4.433, 5.613)	7.164 (4.530, 11.328)	7.03 (4.454, 11.097)
**Sex**				
Female	ref.	ref.	ref.	ref.
Male	1.359 (1.255, 1.472)	1.356 (1.252, 1.469)	1.337 (1.038, 1.721)	1.297 (1.008, 1.671)
**Ethnicity**				
Han	ref.	ref.	ref.	ref.
Others	1.068 (0.939, 1.213)	1.08 (0.950, 1.229)	0.709 (0.331, 1.520)	0.706 (0.33, 1.512)
**Marital status**				
Single	ref.	ref.	ref.	ref.
Married or with partner	0.87 (0.806, 0.938)	0.859 (0.796, 0.927)	0.962 (0.708, 1.307)	0.953 (0.701, 1.295)
Widowed or divorced	0.919 (0.844, 1.001)	0.915 (0.839, 0.997)	1.219 (0.874, 1.701)	1.197 (0.857, 1.672)
Unknown	1.054 (0.773, 1.438)	1.056 (0.774, 1.442)	2.234 (0.787, 6.338)	2.239 (0.789, 6.353)
**Education attainment**				
Tertiary education and above	ref.	ref.	ref.	ref.
Senior high school	1.414 (1.202, 1.664)	1.405 (1.193, 1.654)	1.382 (0.855, 2.235)	1.351 (0.838, 2.176)
Junior high school	1.801 (1.542, 2.104)	1.788 (1.529, 2.090)	1.782 (1.132, 2.803)	1.753 (1.119, 2.746)
Primary school or illiteracy	1.992 (1.691, 2.347)	1.980 (1.680, 2.334)	2.486 (1.551, 3.985)	2.439 (1.526, 3.899)
**Transmission route**				
Homosexual intercourse	ref.	ref.	ref.	ref.
Heterosexual intercourse	1.810 (1.612, 2.032)	1.803 (1.605, 2.024)	4.526 (2.793, 7.335)	4.376 (2.717, 7.048)
Injection drug use	2.355 (2.043, 2.714)	2.337 (2.023, 2.699)	9.101 (5.199, 15.93)	8.607 (4.938, 15.000)
**Occupation**				
Farmer/worker	ref.	ref.	ref.	ref.
Business/service	0.934 (0.872, 1.000)	0.932 (0.869, 0.999)	0.725 (0.574, 0.917)	0.728 (0.575, 0.921)
Professional/manager	0.897 (0.787, 1.023)	0.896 (0.786, 1.022)	0.964 (0.695, 1.337)	0.993 (0.715, 1.379)
Others	0.909 (0.843, 0.979)	0.913 (0.847, 0.984)	0.595 (0.454, 0.781)	0.592 (0.451, 0.779)
**Source of detection**				
Medical institution	ref.	ref.	ref.	ref.
VCT	0.813 (0.752, 0.878)	0.808 (0.747, 0.874)	0.780 (0.610, 0.997)	0.786 (0.615, 1.005)
Special project	0.765 (0.615, 0.951)	0.754 (0.606, 0.939)	0.837 (0.295, 2.378)	0.807 (0.284, 2.294)
Others	0.717 (0.645, 0.797)	0.716 (0.643, 0.797)	1.014 (0.708, 1.453)	1.019 (0.710, 1.464)
**Internal migrant**				
No	ref.	ref.	ref.	ref.
Yes	1.181 (1.111, 1.255)	1.185 (1.115, 1.26)	0.801 (0.559, 1.148)	0.804 (0.561, 1.152)
**CD4 counts, cell/uL**				
≥500	ref.	ref.	-	-
350~500	1.159 (1.062, 1.266)	1.16 (1.061, 1.267)	-	-
200~350	1.651 (1.515, 1.799)	1.652 (1.515, 1.801)	-	-
50~200	3.663 (3.352, 4.002)	3.655 (3.342, 3.996)	-	-
< 50	12.715 (11.509, 14.047)	12.789 (11.564, 14.142)	-	-
**Late testing**				
No	ref.	ref.	-	-
Yes	1.358 (1.275, 1.447)	1.341 (1.258, 1.429)	-	-
**Having an experience in ART**				
No	ref.	ref.	-	-
Yes	0.142 (0.133, 0.151)	0.144 (0.136, 0.154)	-	-
**AHD status**				
Neither late testing nor late ART	-	-	ref.	ref.
Late testing but not late ART	-	-	1.481 (0.892, 2.46)	1.460 (0.879, 2.426)
Late ART but not late testing	-	-	1.690 (1.166, 2.451)	1.638 (1.127, 2.381)
Both late testing and late ART	-	-	1.335 (1.042, 1.710)	1.335 (1.043, 1.707)
**Immediate ART**				
No	-	-	ref.	ref.
Yes	-	-	0.923 (0.755, 1.129)	0.921 (0.753, 1.128)
**Log10 VL, copies/ml**				
Per unit increase	-	-	3.294 (2.005, 5.412)	3.407 (2.085, 5.568)

*HR, Hazard ratio, CI, confidence interval, MD, mean difference, ART, antiretroviral therapy AHD, advanced HIV disease, VL, viral load*.

### Factors Associated With Viral Load and Mortality in the Antiretroviral Therapy Cohort

Supplementary Table 2 showed that PLHIV, in general, received a better improvement once ART initiation [*mean difference* (MD) in log10 scale and 95% *CI* of follow-up the year: −0.272 (−0.278, −0.266)]. Both the late testing and late ART (*MD* = 0.178, 95% *CI*, 0.157–0.199), late testing but not late ART (*MD* = 0.198, 95% *CI*, 0.160–0.236), and late ART but not late testing (*MD* = 0.079, 95% *CI*, 0.029–0.129) lead to higher VL compared to those without late testing or ART. In addition, in general, the clinical decision of ART discontinuation indicates a poorer VL compared to switching ART regimen (*MD* = −0.576, 95% *CI*, 0.629, −0.520). Polypill in the ART regimen does not show significant improvement in VL (*MD* = −0.005, 95% *CI*, −0.064–0.053).

The time-to-event process of joint modeling in the ART cohort showed a similar result to the surveillance cohort in terms of the following factors: age, sex, educational attainment, and transmission route. Of note, internal migrants did not have a higher mortality rate in the ART cohort (Table 3). As to disease stage at entry and ART, late ART was associated with a higher mortality rate. Particularly, PLHIV with both the late testing and late ART had a higher mortality rate (*HR* = 1.335, 95% *CI*, 1.042–1.710) and those with late ART but without late testing were associated with a higher HR of death as well (*HR* = 1.690, 95% *CI*, 1.166–2.451). Immediate ART seemed to be associated with less mortality rate, but was not statistically significant. Higher viral load is a significant predictor of all-cause mortality (*HR* = 3.294, 95% *CI*, 2.005–5.412).

### Subgroup Analysis

The subgroup analysis (Table 3) verified the reliability of the results and the model got consistent estimates with the previous time-extended Cox model for the surveillance cohort and joint modeling for the ART cohort.

## Discussion

This study demonstrated that both the short-term mortality and all-cause mortality among PLHIV in Guangdong Province, China saw a decreasing trend, which could be attributed to the great efforts in control and prevention of HIV and expanding ART coverage in China. In addition, this study provided supportive evidence for the test-and-immediate-treat policy in China and identified that PLHIV with late ART regardless of the AHD status when seeking HIV care were always more vulnerable to mortality. In 2016, China formalized the test-and-immediate-treat policy, expanding the ART recommendation to immediate ART initiation for all the PLHIV regardless of symptoms or CD4 cell count (2). Our tendency analysis supported that the policy has brought appreciable benefits over the decrease of short-term mortality. Although the effect is still unclear in all-cause mortality, the possible longer response lag of the policy about all-cause mortality should be taken into account, thus requiring more time and future research.

One previous study showed immediate ART lower dropout and viral failure rate among PLHIV with relatively high CD4 cell count compared to their counterpart of delayed immediate ART (3). Compared to non-ART, immediate ART can also normalize CD4/CD8 ratio (14) and decrease all-cause mortality (8). However, we did not find evidence that immediate ART brings appreciable health benefits when extending the population to individuals without a CD4 limit. The discrepancy might be attributed to different populations. In line with this study, a cohort in high-income settings also dispelled the idea that immediate ART must bring about myriad health benefits even in a population with relatively low CD4 cell count (4). Thus, we considered in the era of immediate ART, the whole PLHIV might gain less health benefit than expected if PLHIV presented with a late HIV diagnosis or initiated ART at a late disease stage.

Consistent with previous studies, older age, male sex, and lower educational attainment were independent risk factors for mortality (15, 16). Diminished immune function and recovery and higher comorbidity may contribute to an increased risk of death among older PLHIV (16). The relationship between male sex and mortality could be explained by the higher odds of late presentation for care, AHD, and late ART among men (15, 17). Notably, approximately four in five PLHIV were men in this study, which was in line with previous studies (8, 18), highlighting the urgent need to promote early diagnosis and treatment among them. Individuals with lower educational attainment may have less adequate knowledge about HIV/AIDS, leading to a delay HIV diagnosis until symptoms appeared (16). In addition, lower educational attainment was associated with less healthy behaviors and decreased self-assessed health, which may also contribute to a higher risk of mortality (19).

It is a tricky issue of early testing in marginalized populations such as internal migrants who have historically lagged behind HIV care and treatment engagement. Internal migrants generally have lower educational attainment and social position (Supplementary Table 3) and they are over six times risk than that the general population in terms of HIV infection (20). Albeit higher risk, they have less access to health services due to unstable social bonding and other structural social disadvantages (21). This study further states that internal migrants, in general, are more fragile (shown in the surveillance cohort), but if they could access ART and regular VL and CD4 monitoring, they should not have experienced a higher mortality rate. Such health inequity needs to be justified and settled down by the not only health sectors, but also other population management bureaus. It should be encouraged to strengthen the link between government and marginalized populations, enlarge the coverage of health services locally, and initiate ART once diagnosed to prevent late testing and delayed ART.

As an indispensable part of the HIV care continuum, VCT is flattening the HIV-related knowledge gap when high-risk people come to clinics, reducing the possible late testing and late ART. However, it is hard to touch some subpopulations in genuine need through regular VCT purveyed at healthcare centers or the Centers for Disease Control and Prevention (CDC) (17). We could do further establish mobile VCT at the transport interchange for internal migrant workers to access HIV testing. Also, scaling up the regular VCT to community-based VCT (cVCT) and embedding community mobilization, especially in the high-risk community, can mitigate the stigmas and discrimination against HIV counseling services, encourage HIV disclosure and detection, extend the coverage of HIV testing, and discourage high-risk behaviors in the communities (22, 23). Apart from direct benefit, such community-based intervention also trained a lot of volunteers and social workers (23) who in the future could continue their function in society.

More than one-third of people were aware of HIV infection in medical institutions in this study. A potential strategy to increase HIV testing coverage is the opt-out provider-initiated HIV testing and counseling (PITC), which has been demonstrated to be cost-effective in setting with high HIV prevalence. In this study, we encourage providing PITC service, especially at sexually transmitted infection (STI) clinics. VCT and PITC can improve HIV testing at the population level. Another economical and effective way to pinpoint high-risk persons is tracing those with confirmed HIV diagnoses to their partners. In 2020, the UNAIDS reported more than half of new diagnosed HIV infections that were identified by their sexual partner (1) and assisted partner notification (APN) service is a more effective approach compared to passive notification, allowing health staff to directly communicate with the partner of PLHIV and assuring the partner with higher uptake of HIV testing (24). Though PLHIV might be unwilling to receive the service due to fears of relationship repercussions and stigmas (25), in general, it is few adverse events after APN service.

After HIV diagnosis, initiating ART and retention become our primary concerns. Once a patient starts ART, we should be committed to keeping them in ART, preventing them from treatment discontinuation. Adverse effects of ART were one of the obstacles to treatment continuation and we consider that PLHIV (26), in general, might get less harm from ART switching rather than discontinuation when necessary to modify the undergoing ART therapy due to adverse effects from drugs, according to our results of ART discontinuation with a higher risk of elevated VL. Apart from adverse effects, adherence of patients also hinges on the convenience of treatment. Single-tablet regimens might provide a valid strategy to deal with the problem of missing doses of HIV medication and improve the prognosis (27). In this study, an educated guess is that benefit of a single-tablet regimen identified by other research can be generalized in China. However, we had not have enough samples on single-tablet regimens since single-tablet regimens are not on the list of free ART drugs. Alternatively, we explored the relationship between polypill regimen and log_10_VL, but the result is insignificant, leaving the hypothesis uncertain for future researchers.

Community-based organizations (CBOs) could play more critical roles in the HIV management package. In our data, a large proportion of PLHIV discontinued ART due to their unwillingness rather than adverse effect (Supplementary Table 4). Adherence should not entirely rely on tertiary hospitals and decentralizing HIV care can release the burden of tertiary hospitals and improve the health of PLHIV. A systematic review demonstrated the improvement of ART retention by initiating ART at the hospital and continuing in CBO (28), which received better satisfaction from PLHIV in respect of clinician spending time and HIV knowledge (29). Moreover, continuing ART at home provided by communities might be an alternative, which is performed and at clinics by professional health workers (28). Trained (peer) volunteers and social workers could be fully functional in communities, ameliorating social disadvantages for those with low educational attainment and providing support for newly-diagnosed PLHIV or those experiencing stressful and traumatic life events such as divorce or widowhood, helping them overcome the difficulties and encouraging them in retention (30).

The UNAIDS appealed for “*communities to make the difference*” on the 2019 world AIDS day (31). China is stepping into supporting community service, for example, the China AIDS fund for a non-governmental organization (NGO) funded millions of Yuan for local NGOs in Yunnan, Guangxi, and other provinces for HIV prevention and control by 2018 (2). Such support was inspiring, but we considered that government can do more to improve the initiation in communities. AIDS education campaigns should be continued and strengthened in the community to improve the awareness of AIDS and the risk perception of HIV infection (17). We need to enable the community with rapid HIV testing capacity to purvey service in multiple settings for the local, namely, home-based self-testing, outreach and primary care center-based rapid testing, and counseling (32). Either facility-based HIV testing or HIV self-testing could be combined with the basic public health services in the community to expand HIV testing coverage (17). These comprehensive services are the key to decreasing health inequity and achieving sustainable development goals of “ending the AIDS epidemics” by 2030 (1). We still expect further promotions in community service and consider it can make the HIV community different and deserved to get more attention, support and funding from the government and society.

Several limitations might bring a negative impact on the validity of our result. First, we cannot eliminate the unmeasured confounder and biases in a retrospective observational study. Second, many PLHIV were not eligible for individual analysis in this study due to a lack of sufficient laboratory information and regular follow-up. Thus, our result could be only extrapolated to the PLHIV by adhering to the cohort and performing the regular medical check.

In conclusion, this study justified the concern about late testing/ART under the test-and-immediate-treat policy. Though the movement toward immediate ART in China is inspiring, late testing/ART has been impairing the effect of immediate ART greatly. Late testing/ART could be improved by comprehensive HIV testing programs. Partial difficulties of adherence to ART could be accused with adverse effects and inconvenience. However, currently, highlighting community HIV support would be more needed, which favors the overall HIV care cascade by decreasing the stigmas and discrimination of HIV testing, encouraging HIV testing, and keeping PLHIV in ART.

## Data Availability Statement

The original contributions presented in the study are included in the article/Supplementary Material, further inquiries can be directed to the corresponding authors.

## Ethics Statement

The studies involving human participants were reviewed and approved by the Ethics Committee of Guangdong Pharmaceutical University. Written informed consent to participate in this study was provided by the participants' legal guardian/next of kin.

## Author Contributions

HJ and YL contributed equally to this study and contributed to the study concept and design. HJ had full responsibility for the finished article, had access to the data, and controlled the decision to publish. QC, JLiu, XF, FY, QL, JbL, ZT, JLi, KL, and YYan contributed to data collection, data interpretation, and literature search. QC performed the statistical analysis and drafted the manuscript. HJ, YL, and YYang critically revised the manuscript. All the authors have approved the final version of the manuscript.

## Funding

This research was funded by the Philosophy and Social Science of Guangdong Province (GD21YSH08) and the National Natural Science Foundation of China (81703282).

## Conflict of Interest

The authors declare that the research was conducted in the absence of any commercial or financial relationships that could be construed as a potential conflict of interest.

## Publisher's Note

All claims expressed in this article are solely those of the authors and do not necessarily represent those of their affiliated organizations, or those of the publisher, the editors and the reviewers. Any product that may be evaluated in this article, or claim that may be made by its manufacturer, is not guaranteed or endorsed by the publisher.
