# Supplementary material for: Effect of Late Testing and Antiretroviral Treatment on Mortality Among People Living With HIV in the Era of Treat-All in Guangdong Province, China, 1992–2018: A Cohort Study
Source: Front Public Health. 2022 Jul 8;10:851117. doi: 10.3389/fpubh.2022.851117 (PMC9304903; doi:10.3389/fpubh.2022.851117)
Supplement: Supplementary file 1 [file Table_1.docx]

**Content**

**Supplementary table 1: Viral load distribution at the entry and end of cohort stratified by immediate ART**

**Supplementary table 2: Summarization of educational attainment, social position and source of HIV detection stratified by internal migration status**

**Supplementary table 3: coefficient about the association between log10 viral load and variables in ART cohort**

**Supplementary table 4: Summarization of the reason for ART discontinuation decision**

**Supplementary table 1: Viral load distribution at the entry and end of cohort stratified by immediate ART**

|  | **No immediate ART** | **Immediate ART** | **Proportion difference^1^** | **P-value^2^** |
| --- | --- | --- | --- | --- |
|  | **(N=14352)** | **(N=11935)** | **(N=26287)** |  |
| **VL at entry to ART cohort, copies/mL, %** | - | - | - | 0.0192 |
| ≤400 | 7760 (54.1) | 6370 (53.4) | 0.696 % (0.618) |  |
| >400 | 6592 (45.9) | 5565 (46.6) |  |  |
| **Most recent viral load, copies /mL, %** | - | - | - |  |
| ≤400 | 13650 (95.1) | 11455 (96.0) | -0.869 % (0.254) |  |
| >400 | 702 (4.9) | 480 (4.0) |  |  |

1: Proportion difference refers to the >400 VL at entry/most recent proportion difference between no immediate ART and Immediate ART group

2: P-value refers to the difference of proportion difference

Normal approximation was used for P-value calculation.

VL=viral load, ART=anti-retroviral therapy

**Supplementary table 2: coefficient about the association between log_10_ viral load and variables in ART cohort**

|  | **MD (95% CI) of Log10VL in the ART cohort (2005-2018)** | **MD (95% CI) of Log10VL in the ART cohort (2009-2018)** |
| --- | --- | --- |
| **Years of follow-up** |  |  |
| Per year increase | -0.272 (-0.278, -0.266) | -0.278 (-0.284, -0.273) |
| **Age, yrs** |  |  |
| 15~29 | ref. | ref. |
| 30~44 | 0.024 (0.001, 0.048) | 0.023 (0, 0.047) |
| 45~59 | 0.056 (0.022, 0.09) | 0.053 (0.019, 0.087) |
| 60~ | 0.076 (0.026, 0.127) | 0.082 (0.032, 0.133) |
| **Sex** |  |  |
| Female | ref. | ref. |
| Male | 0.106 (0.078, 0.134) | 0.11 (0.082, 0.138) |
| **Marital status** |  |  |
| Single | ref. | ref. |
| Married or with partner | -0.064 (-0.09, -0.038) | -0.065 (-0.091, -0.039) |
| Widowed or divorced | -0.017 (-0.049, 0.015) | -0.022 (-0.054, 0.01) |
| Unknown | -0.279 (-0.434, -0.124) | -0.277 (-0.432, -0.122) |
| **Education attainment** |  |  |
| Junior high school | ref. | ref. |
| Primary school or illiteracy | 0.061 (0.027, 0.094) | 0.06 (0.026, 0.094) |
| Senior high school | 0.027 (0.003, 0.051) | 0.024 (0, 0.048) |
| Tertiary education and above | 0.033 (0.007, 0.058) | 0.033 (0.007, 0.058) |
| **Transmission route** |  |  |
| Homosexual intercourse | ref. | ref. |
| Heterosexual intercourse | -0.028 (-0.051, -0.005) | -0.028 (-0.051, -0.005) |
| Injection drug use | 0.258 (0.207, 0.308) | 0.248 (0.197, 0.299) |
| **AHD status** |  |  |
| Neither late testing nor late ART | ref. | ref. |
| Late testing but not late ART | 0.079 (0.029, 0.129) | 0.085 (0.035, 0.134) |
| Late ART but not late testing | 0.198 (0.160, 0.236) | 0.203 (0.165, 0.241) |
| Both late testing and late ART | 0.178 (0.157, 0.199) | 0.173 (0.152, 0.193) |
| **Clinical decision** |  |  |
| ART discontinuation | ref. | ref. |
| Keeping original ART regimen | -0.673 (-0.727, -0.618) | -0.677 (-0.732, -0.623) |
| Treatment regimen switching | -0.575 (-0.629, -0.52) | -0.579 (-0.634, -0.524) |
| **Polypill in ART regimen** |  |  |
| No | ref. | ref. |
| Yes | -0.005 (-0.064, 0.053) | -0.007 (-0.066, 0.051) |

MD: Mean difference

**Supplementary table 3: Summarization of educational attainment, social position and source of HIV detection stratified by internal migration status.**

|  | **Overall** | **No internal migrant** | **Internal migrant** | **P-value** |
| --- | --- | --- | --- | --- |
|  | **(N=49289)** | **(N=36667)** | **(N=12622)** |  |
| **Educational attainment, %** | - | - | - | <0.0001 |
| Tertiary education and above | 8752 (17.8) | 6913 (18.9) | 1839 (14.6) |  |
| Senior high school | 10577 (21.5) | 8268 (22.5) | 2309 (18.3) |  |
| Junior high school | 20073 (40.7) | 14580 (39.8) | 5493 (43.5) |  |
| Primary school | 8810 (17.9) | 6117 (16.7) | 2693 (21.3) |  |
| Illiteracy | 1077 (2.2) | 789 (2.2) | 288 (2.3) |  |
| **Occupation, %** | - | - | - | <0.0001 |
| Farmer/worker | 9764 (19.8) | 7003 (19.1) | 2761 (21.9) |  |
| Business/service | 20558 (41.7) | 15490 (42.2) | 5068 (40.2) |  |
| Professional/manager | 2135 (4.3) | 1753 (4.8) | 382 (3.0) |  |
| Others | 16832 (34.1) | 12421 (33.9) | 4411 (34.9) |  |
| **Source of HIV detection, %** | - | - | - | <0.0001 |
| Medical institution | 27765 (56.3) | 21050 (57.4) | 6715 (53.2) |  |
| Voluntary Counselling and Testing | 13216 (26.8) | 10174 (27.7) | 3042 (24.1) |  |
| Special project | 2587 (5.2) | 2038 (5.6) | 549 (4.3) |  |
| Others | 5721 (11.6) | 3405 (9.3) | 2316 (18.3) |  |

P-value was calculated by chi-square testing

**Supplementary table 4: Summarization of the reason for ART discontinuation decision**

|  | **Overall (N=951)** |
| --- | --- |
| **Reason for ART discontinuation, %** |  |
| Treatment failure | 2 (0.2) |
| Multiple drug interaction | 8 (0.8) |
| Adverse effect | 89 (9.4) |
| Economical difficulty | 5 (0.5) |
| Unwillingness of adherence | 399 (42.0) |
| Others | 411 (43.2) |
| Multiple drug interaction + Unwillingness of adherence | 1 (0.1) |
| Adverse effect + Multiple drug interaction | 1 (0.1) |
| Adverse effect + Unwillingness of adherence | 6 (0.6) |
| Adverse effect + Others | 4 (0.4) |
| Unwillingness of adherence + Others | 24 (2.5) |
| Adverse effect + Multiple drug interaction + Unwillingness of adherence | 1 (0.1) |

ART=anti-retroviral therapy
